# Targeting Tumor-Associated Macrophages for Imaging

**DOI:** 10.3390/pharmaceutics15010144

**Published:** 2022-12-31

**Authors:** Jiahao Hu, Xiaoling Xu, Yongzhong Du

**Affiliations:** 1 Institute of Pharmaceutics, College of Pharmaceutical Sciences, Zhejiang University, Hangzhou 310058, China; 2 Shulan International Medical College, Zhejiang Shuren University, Hangzhou 310015, China

**Keywords:** tumor-associated macrophages, targeting, imaging, theranostics, nanoprobes

## Abstract

As an important component of the tumor immune microenvironment (TIME), tumor-associated macrophages (TAMs) occupy a significant niche in tumor margin aggregation and respond to changes in the TIME. Thus, targeting TAMs is important for tumor monitoring, surgical guidance and efficacy evaluation. Continuously developing nanoprobes and imaging agents paves the way toward targeting TAMs for precise imaging and diagnosis. This review summarizes the commonly used nanomaterials for TAM targeting imaging probes, including metal-based nanoprobes (iron, manganese, gold, silver), fluorine-19-based nanoprobes, radiolabeled agents, near-infrared fluorescence dyes and ultrasonic nanobubbles. Additionally, the prospects and challenges of designing nanomaterials for imaging and diagnosis (targeting efficiency, pharmacokinetics, and surgery guidance) are described in this review. Notwithstanding, TAM-targeting nanoplatforms provide great potential for imaging, diagnosis and therapy with a greater possibility of clinical transformation.

## 1. Introduction

Cancer ranks as one of the leading causes of death in modern society around the world and is also a major obstacle to the improvement of the human lifespan. In 2020, 19 million new cancer cases were reported worldwide (18.1 million cases, excluding nonmelanoma skin cancer) and almost 10 million cancer deaths (9.9 million, excluding nonmelanoma skin cancer) [1]. Because of the deadly health and safety threats from cancer, effective and economical treatments are urgently needed. Currently, the mainstream clinical treatment approaches for cancer include surgery, radiotherapy, chemotherapy and immunotherapy, followed by some adjuvant therapies (microwave diathermy, interventional therapy, radiofrequency ablation, ultrasonic therapy and laser therapy [2]). Although these therapeutic regimens have achieved some positive outcomes, various limitations exist. (1) Both surgery and radiotherapy approaches require an accurate definition of the tumor boundary [3,4,5]. (2) Therapeutic monitoring after chemotherapy agent exposure is inadequate. Meanwhile, it has a good response on primary and metastatic lesions, but it often causes serious toxicity and side effects, leading to a decline in drug tolerance, drug resistance and consequently tumor recurrence.

Undoubtedly, most treatment failure and cancer recurrence are associated with the complicated tumor microenvironment [6]. It is a hypoxic and acidic condition with abundant production of immune-suppressive metabolites [7]; many immune cells, stromal cells and blood vessels; and an extracellular matrix. Notably, the composition and proportion vary among different tumor types and different stages of tumor progression, further altering the immune phenotypes of tumor cells [6]. Immune cells in the tumor microenvironment mostly include T cells, B cells, monocyte macrophages, NK cells and dendritic cells [8]. Among them, tumor-associated macrophages (TAMs) play an important role in the immune microenvironment. The infiltration of TAMs is closely related to the poor survival rate of patients with solid tumors [9]. With potent polarizable properties, macrophages are usually classified into the classic-activation M1 type and anti-inflammatory M2 type. M2 macrophages are considered to be the most common type of TAM and can be further subdivided into the M2a phenotype (induced by IL-4 or IL-13), M2b phenotype (with high IL-10 and low IL-12) and M2c phenotype (with low TNF-α) [10]. The overexpression of CSF-1 and epithelial mesenchymal transformation (EMT) assemble massive TAMs at the tumor margin [11,12]. Moreover, it was found that macrophages and other stromal cells may form desmoplastic structures to prevent the infiltration of cytotoxic lymphocytes (CTLs) into the tumor core. These results indicate that TAMs play an essential role in edge identification between tumor and normal cells, as well as therapeutic evaluation. Hence, targeting TAMs can be meaningful in imaging and therapy, including profiling tumor boundaries for directing surgery, biopsies and radiotherapy, examining the ratio of M1 type and M2 type for assessing treatment responses and obtaining prognostic information [13,14].

**Figure 1:**
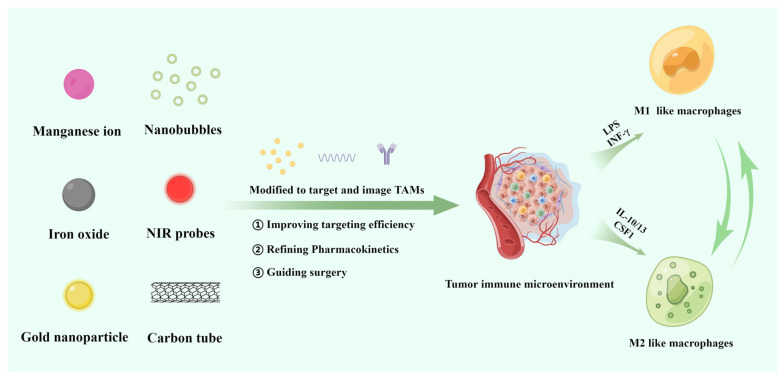
Schematic diagram of materials of tumor-associated macrophages targeting strategy.

TAMs-targeted imaging probes combine the existing imaging technology and TAM-targeted modification to target tumor-associated macrophages through the modification of mannose, polypeptide and polysaccharide against the receptors, such as CD206 (human mannose receptor), TREM2 (triggering receptor expressed on myeloid cells 2), legumain and scavenger receptor-A overexpressed by TAMs [15]. This review summarizes TAM-targeting construction materials in preclinical and in vitro experimental research, including metal-based nanoprobes (iron, manganese, gold, silver), fluorine-19-based nanoprobes, radiolabeled agents, near-infrared fluorescence dyes and ultrasonic nanobubbles, and then analyzes the principles of TAM-targeting strategies in guiding operations (Figure 1).

## 2. Nanoprobes for TAM Imaging and Therapy

### 2.1. Metal-Based Nanoprobes

#### 2.1.1. Iron-Based Nanoprobes

Imaging of TAMs using MRI usually takes metal elements as the core coating with the targeting moiety and responsive chemical bonds or enzymes [16]. As frequently used diagnostic molecules, Fe-based MRI agents obtain sensitive responses to magnetic environments, even in the intracellular environment, after being engulfed by macrophages. Superparamagnetic iron oxide materials can further improve the ability of magnetic resonance imaging by reducing the intensity of the T2 signals in the tissues that absorb the contrast agent.

To track the imaging of glioblastoma (GBM) tumor-associated macrophages (TAMs), Runze Yang [17] et al. recently demonstrated that using iron oxide nanoparticles (USPIOs) alone in MRI potently tracked the monocyte–macrophage system. They confirmed that both quantitative susceptibility mapping (QSM) and R2* mapping MRI after injecting USPIO were able to reflect iron- and deoxyhemoglobin-induced MRI changes. The obtained data indicated the enhancement of monocyte infiltration in the GBM tumor environment under nicotinic acid treatment, and USPIO-mediated MRI was more sensitive than traditional MRI anatomical imaging in the evaluation of the treatment effect.

**Figure 2:**
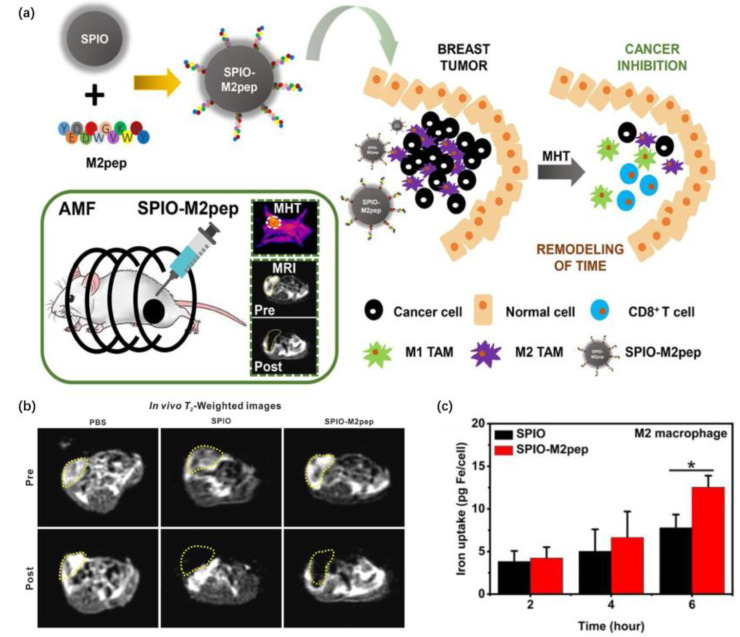
(**a**) Schematic of the preparation of SPIO-M2pep and the mechanism of SPIO-M2pep in M2 TAM-targeted and MRI-guided magnetic hyperthermia therapy of breast cancer. (**b**) MR imaging of orthotopic breast tumor mice before and 30 min after injection of SPIO or SPIO-M2pep (tumor: yellow dashed line) (**c**) Iron uptake in M2 macrophage cells treated with SPIO and SPIO-M2pep for different incubation times (25 μg mL^−1^, n = 3). *p* values less than 0.05 are considered significant and marked with * [19]. Copyright 2021, Elsevier.

The surface modification of the iron oxide nanoplatform effectively improves the targeting ability for TAMs. Li et al. [18] designed PEG-b-AGE polymer-coated iron oxide nanoparticles targeting the mannose receptor of M2-like macrophages. Showing robust M2-like macrophage imaging and antibiofouling ability, this nanoplatform revealed the potential to study the dynamics of TAMs and the tumor microenvironment. Wang et al. [19] reported M2 macrophage-targeted peptide (sequence: YEQDPWGVKWWY)-functionalized superparamagnetic iron oxide (SPIO) nanoparticles that possessed M2 macrophage targeting and magnetic resonance imaging abilities. In terms of the relativity coefficient, SPIO was as effective as a commercial MR contrast agent. In vivo experiments confirmed the high efficiency of the M2-targeting technique because the average tumor volume of the SPIO-M2pep + alternating magnetic field (AMF) group (265.07 mm^3^) was much smaller than that of the SPIO + AMF group (1319.50 mm^3^). Moreover, by remodeling the tumor immune microenvironment by replenishing cytokines and related immune cells, SPIO also exhibited depletion and repolarization of M2 macrophages; thus, the entire nanoprobe was described as a multi-stratal diagnosis and treatment system (Figure 2a). Assessing the imaging ability of modified SPIO by qualifying the macrophage cellular iron concentration after 6 h of coculturing with SPIO-M2pep, the average iron concentration in the SPIO-M2pep-treated group was 12.54 pg/cell, which was significantly higher than that in the normal SPIO-treated group (7.97 pg/cell) (Figure 2c). The in vivo imaging results implied that SPIO-M2pep had a relaxation coefficient (149 mM^−1^s^−1^) that was comparable to that of Resovist (143 mM^−1^s^−1^), a commercial contrast agent composed of very small (5.8 ± 2.5 nm) superparamagnetic iron oxide (USPIO) with a carboxydextran coating.

In recent years, superparamagnetic iron oxide nanoparticles have also been used in magnetic particle imaging (MPI) positioning according to the signal generated by SPIO in a free magnetic field. In contrast to MRI, MPI provides quantitative information on in vivo iron labeling of macrophages in a 4T1 mouse model, indicating that MPI may serve as an adjunct to other imaging techniques. MPI technology can supplement the quantitative information of local iron [20]. Combined with MRI, it can eliminate some false-positive signals in the tumor boundary determination of targeted macrophages.

Regarding diagnosis and treatment integration, the diversity of Fe-based imaging probe preparation methods endows iron-related nanoparticles with potential as drug carriers for loading tumor-killing drugs or microenvironment-regulating cytokines. Additionally, the discoveries of the iron oxide magnetocaloric effect and ferroptosis signaling pathway strengthened the antitumor function of Fe-based nanoparticles [21]. The MRI imaging ability of combined ferric oxide as an evaluation of the efficacy of related macrophage-targeted antitumor agents has become a possible future of the integrated process of diagnosis and treatment. To trigger an in situ catalytic cascade reaction to generate abundant ROS and reeducate antitumor immunity, a nanosystem combining superparamagnetic iron oxide nanocrystals and nitric oxide donors was fabricated [22]. Releasing NO, turning TAMs to the M1 phenotype and promoting partial tumor infiltration of effector T cells, the nanoplatform has the potential to track and evaluate the effect by MRI.

**Figure 3:**
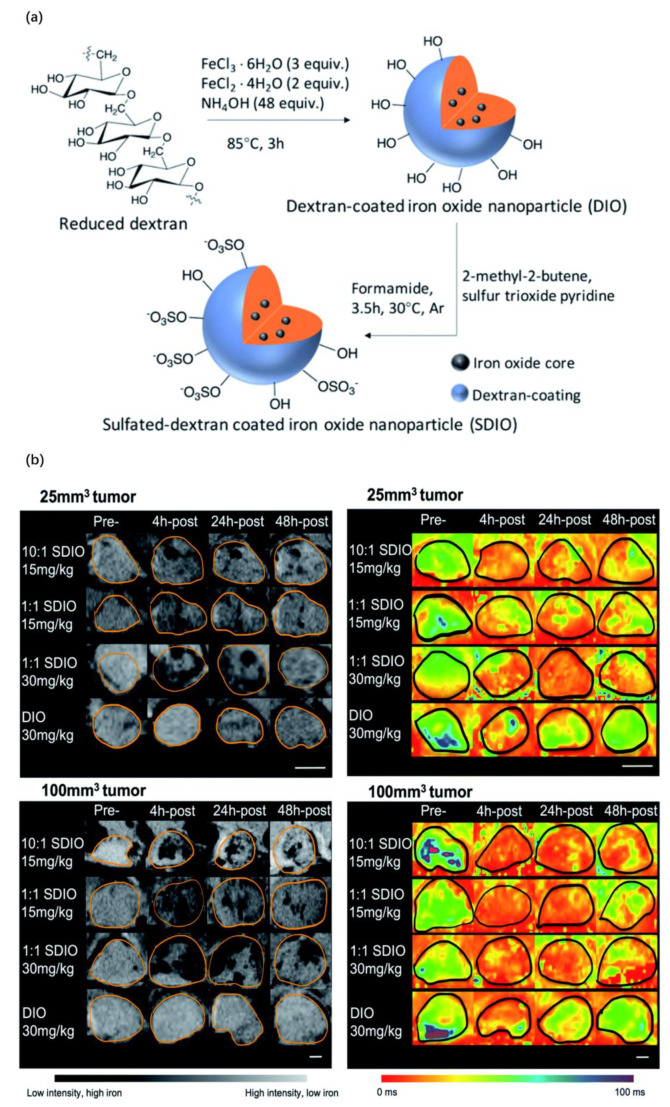
(**a**) Synthetic route of SDIO/DIO. (**b**) Representative zoomed in MR images and parametric T2* maps for 25 mm^3^ tumor and 100 mm^3^ tumor models injected with either 15 mg Fe kg^−1^ 10:1 SDIO, 15 mg Fe kg^−1^ 1:1 SDIO, 30 mg Fe kg^−1^ 1:1 SDIO, or 30 mg Fe kg^−1^ DIO at Pre-, 4 h-post, 24 h-post, or 48 h-post injection time point (tumor margins are outlined in orange) [23]. Copyright 2022, The Royal Society of Chemistry (Reprinted from Ref. [23] under the terms of the Creative Commons CC BY license).

Additionally, sulfated dextran-coated iron oxide nanoparticles (SDIO) can target macrophage scavenger receptor A (SR-A, also known as CD204) and obtain better retention and imaging efficacy [23]. Likewise, SDIO with 1:1 surface sulfation sites and hydroxyl sites can promote the polarization of macrophages to the M1 phenotype to a certain extent by stimulating macrophages to secrete more TNF-α and IL-6, which may inspire the regulation of the tumor microenvironment. Similarly, this process can be observed by MRI. In research on probe uptake by bone marrow-derived macrophages (BMDMs), the absorption efficiency of 10:1 SDIO was potently higher than that of 1:1 SDIO, the nontargeting DIO and the control group. This ratio represents the quantity ratio of sulfur and hydroxyl groups during synthesis (Figure 3a). The in vivo imaging contrast results showed that SDIO preferentially accumulated in the tumor region under the same iron injection dose (Figure 3b).

#### 2.1.2. Manganese-Based Nanoprobes

Manganese ions have powerful MR imaging ability because the five unpaired electrons in their 3d orbitals can produce a large magnetic moment and cause vibration relaxation of surrounding water molecules, indicating that MnO NPs are a great candidate for T1-MR developing agents [24,25]. However, Mn^2+^ complexes exhibited short blood circulation times and brain assembly toxicity. To avoid the abovementioned detrimental effects, Luo et al. created a bioactivated in vivo assembly (BIVA) magnetic resonance (MR) probe called TSP (mannose-Lys-Lys-Lys-Lys-Arg-Arg-Lys-Ppa[Mn]). Mannose-modified nanoparticles-targeted TAMs and mediated molecular internalization [26]. The polypeptide sequence has the cleavage site of cathepsin B to achieve efficient enzyme response cleavage. The chlorophyll derivative part chelating the paramagnetic contrast agent Mn^2+^ is the assembly part, which is induced to form J-type molecular accumulation after enzyme digestion. The intracellular dynamic cathepsin B cutting and dynamic assembly of the residues promoted the dynamic aggregation process of the TSP group within 1–8 h. Additionally, the α_agg_ value (aggregation degree) between 8 and 12 h inside the cells was stable at 71.2 ± 3.3%. Furthermore, the α_agg_ value in the female BALB/C nude mouse model was 55.6%. With the lowest detection limit being 10^–4^ M, TSP prolonged the time window of detection (the best MR imaging window at 4 h postinjection) and enhanced the T1 signal.

Furthermore, manganese oxide nanoparticles, which can also carry chemotherapy drugs and monitor the distribution of drugs after administration through MRI, is a great nanoplatform for responding to hypoxia and mildly acidic pH in the TIME [27]. In terms of combination with treatment, combining the imaging ability of MRI imaging agents and the tumor-killing ability of chemotherapy drugs can better evaluate the penetration and antitumor effect of agents at tumor sites. Song et al. [28] coated manganese dioxide colloidal nanoparticles with polyelectrolyte poly(allylamine hydrochloride) (PAH) and modified mannan on the surface to form a nanoplatform for enhanced tumor MRI and targeting macrophages. Combining MnO_2_/H_2_O_2_ to alleviate the hypoxic microenvironment and suppress the expression and secretion of HIF-1α and VEGF, Man-HA-MnO_2_ created Mn^2+^ in an acidic environment to enhance MRI signal intensity. Accumulating in the tumor site, this nanoplatform also enhanced the T1&T2 MRI signals in a dose-dependent manner. Furthermore, MRI was performed on 4T1 tumor mice after intravenous administration of 13.2 mg/kg Man HA MnO_2_ NPs or saline. The obtained results showed a decrease in registered T2 values within tumors, achieving values of 29.8% and 58.6% (with respect to values pre-administration) on day 3 and day 7, respectively, after being treated with Man-HA-MnO_2_ NPs. With the oxygen concentration remarkably increasing, the effect of chemotherapy was further enhanced.

Lim et al. [29] proposed a novel TAM remodeling nanocomposite, hyaluronic acid (HA) and PLR-coated manganese dioxide (MnO_2_) nanoparticles (hpMNPs), releasing manganese ions as a response to high concentrations of glutathione (GSH) in the TIME. The expression of iNOS in macrophages was subsequently increased by free manganese ions, reinforcing its potential regarding the enhancement of signals in T1-weighted images during MRI. NO is a multifunctional signaling molecule that plays an important role in reducing tumor proliferation. Song et al. [30] developed a multifunctional rattle-structured nanotheranostic with hollow mesoporous silica coating UCNP and DOX as the effector molecule. The highlight of this work was the real-time nanosystem imaging of MRI and precise control of drug release. In the acidic microenvironment caused by hypoxia, MnO_2_ degraded to Mn^2+^, which improved the signal intensity of T1-MRI. Nanodiagnosis and treatment technology combining hypoxia-driven T1-MRI with synergistic chemotherapy has great potential in the intelligent diagnosis, individualized treatment and good prognosis of solid tumors in the future.

#### 2.1.3. Gold-Based Nanoprobes

**Figure 4:**
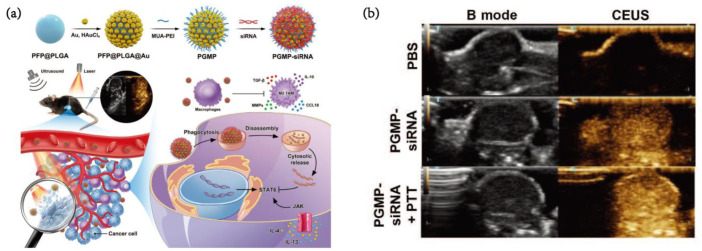
(**a**) Schematic illustration of the preparation and antitumor strategy of PGMP-siRNA NPs. (**b**) In vivo CEUS imaging and B mode of nanoprobes [32]. Copyright 2022, Springer *Nature*.

Gold nanoparticles (AuNPs) have piqued great interest in the diagnosis and therapy of cancers owing to their intrinsic properties. Due to the plasticity and ductility of gold, it is easy to shape different nanostructures. The surface chemical modification or polymer coating of the formed nanostructures provides gold nanoparticles with better biocompatibility and cycle stability. Meanwhile, gold nanoparticles have been used to varying degrees in fluorescence imaging, X-ray imaging, MRI, positron emission tomography/computed tomography (PET/CT) and photoacoustic imaging (PAI) and are powerful and potential imaging materials [31]. In the TAM targeting of gold nanoprobes, Lv et al. [32] designed gold-based nanocarriers (PGMP-small interfering RNA (siRNA) nanoparticles (NPs)) containing STAT6 siRNA for PTT (photothermal therapy) and gene therapy of non-small cell lung cancer (NSCLC). PFP@PLGA(poly(lactic-co-glycolic acid) nanodroplets were prepared by the solvent emulsion/evaporation method and then conjugated with MUA-PEI to carry siRNA. Finally, PFP@PLGA@Au@MUA-PEI (PGMP) nanoparticles were fabricated (Figure 4a). Meanwhile, in contrast-enhanced ultrasound (CEUS) imaging in vivo, the PGMP+PTT group showed more details in the presence of the 808 nm NIR laser than the contrast group in the imaging effect (Figure 4b). After 10 min of 808 nm NIR laser continuous illumination, the tumor-site temperature of the PGMP-siRNA-treated mice increased from 36.2 to 54.3 °C without tumor recurrence, while that of the PBS-treated mice increased from 36.5 to 38.9 °C.

By serving OVA as a model antigen, Sun et al. [33] effectively improved the macrophage phagocytosis of glycol-chitosan-coated gold nanoparticles (GC-AuNPs). GC-AuNPs exhibited superior performance in CEUS/PA imaging of lymph nodes and were able to accelerate the process of antigen presentation.

#### 2.1.4. Silver-Based Nanoprobes

**Figure 5:**
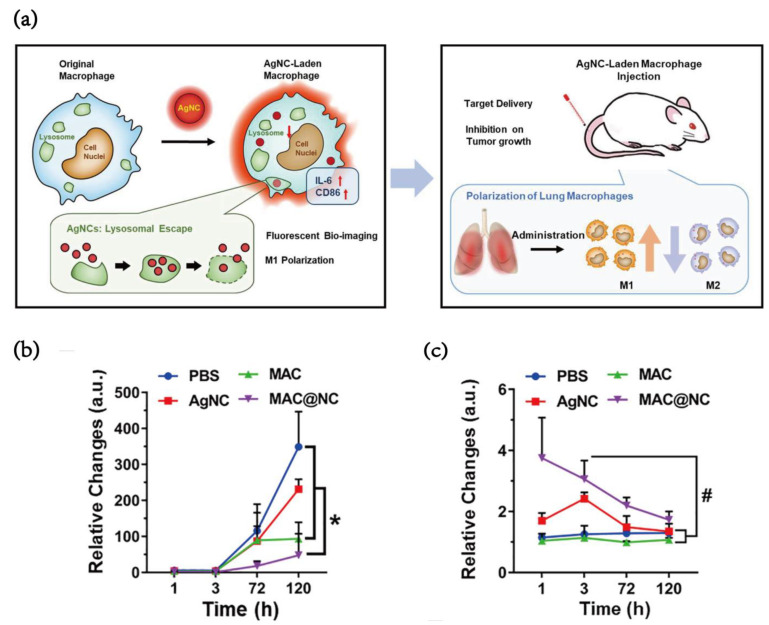
(**a**) Schematic diagram of silver nanoparticles-regulating macrophages. (**b**) Quantification of metastatic tumor growth in the lungs over time, (**c**) Relative changes of AgNC signals relative to the untreated control, where i.v. injection of 4T1-LG12 cells was performed (1.0 × 105 cells/mouse, n = 5), followed by treatments with PBS, AgNC, MAC and MAC@NC for 18 d Statistical analysis between groups: (*) *p* < 0.05 and (#) *p* < 0.001. [35]. Copyright 2022, Elsevier.

Silver nanoparticles (AgNPs) have been increasingly used in pharmaceuticals and medicine due to their unique chemical, physical and biological intrinsic properties [34]. Recently, Yan et al. [35] reported a novel type of AgNCs with super fluorescence stability after being devoured by macrophages for 48 h. AgNCs, with excitation and emission wavelengths of approximately 500 and 700 nm, respectively, were taken up by macrophages and then triggered the proinflammatory responses of macrophages by the activation of Toll-like receptor 4 (TLR4). Because of the lysosomal escape effect, AgNC obtains stable and permanent fluorescence in vivo. This result demonstrated that 24 μg of AgNCs was taken up by 2.0 × 10^6^ macrophages, which means that approximately 1.2 × 10^−5^ μg of AgNCs was taken up by a single macrophage. Displaying an effective targeting tendency toward metastatic tumors, AgNCs markedly inhibited tumor growth and modulated the tumor immune microenvironment. Moreover, Pal et al. [36] reported that AgNC-induced oxidative stress modulates TAMs from an M2 to an M1 phenotype (Figure 5a). Moreover, macrophages containing AgNCs formed after coculturing with AgNCs had a significant inhibitory effect on the lung metastasis of tumor cells (Figure 5b). The red fluorescence signal from MAC@CN could be observed in the lung 120 h after treatment (Figure 5c).

### 2.2. Fluorine-19-Based Nanoprobes

The ^19^F isotope is not naturally present in the body, and the ^19^F signal is completely independent of the hydrogen signal normally used for routine MRI (^1^H MRI) [37]. Both iron oxide (USPIO) and perfluorocarbon (PFC) are classic MRI contrast media [38]. However, there is some differentiation between USPIO and PFC during practical application. USPIO showed high detection sensitivity, while PFC performed better in imaging effect in long-term circulation., which could remedy USPIO imaging loss of partial time. Zambito et al. [39] designed a fluorine-19(^19^F) PLGA MR imaging agent-encapsulating perfluoro-15-crown-5-ether (PFCE) as an MRI contrast agent with mannose modification on its surface. Targeting M2 macrophages in humanized mice bearing breast cancer as a tumor model, ^19^F-PLGA-NPs showed reassuring biosafety. Notwithstanding that it is difficult for this probe to avoid a high background signal from the liver, the concentration of PFCE could be quantified.

In the meantime, the microenvironment conditions of different tumors and tumors in different periods are also very different, which requires the imaging probe design to address a differentiated tumor treatment situation [40]. People used ^19^F MRI in preclinical models of gliomagenesis and breast-to-brain metastasis to confirm the technical feasibility of longitudinal and noninvasive imaging in vivo with a clinical scanner at high spatial resolution. Multispectral ^19^F MRI can recognize the different spatial and temporal niches of TAMs in radiotherapy (RT)-recurrent murine gliomas [30]. The content and composition of TAMs constantly change and adapt during tumor growth and response to treatment [41]. Under these circumstances, ^19^F MRI represents a powerful strategy to track different TAM dynamics in RT recurrent tumors in time and space using PFCE-NP and perfluoro-tert-butylcyclohexane (PFTBH)-NP for multispectral fluorine-19 MRI [42]. The obtained data show that glioma not only changes the ratio of microglioma cells (MGs) to macrophages (MDMs) in response to RT but also alters the spatial distribution and populations of these different cells. It is important to assess whether these microenvironmental niches defined by TAMs have responses to specific treatments. ^19^F MRI can represent a potential MRI-based approach to obtain important information about BBB leakage and heterogeneity. In the glioma recurrence model, the ^19^F signal from PFTBH-NP mainly covered the site of recurrent tumor tissue. At the same time, the researchers also evaluated the spatial rejection of MG and MDM in the tumor after radiotherapy through the multispectral ^19^F MRI strategy. MG and MDM will be farther in space than before radiotherapy.

### 2.3. Radiolabeled Agents

**Figure 6:**
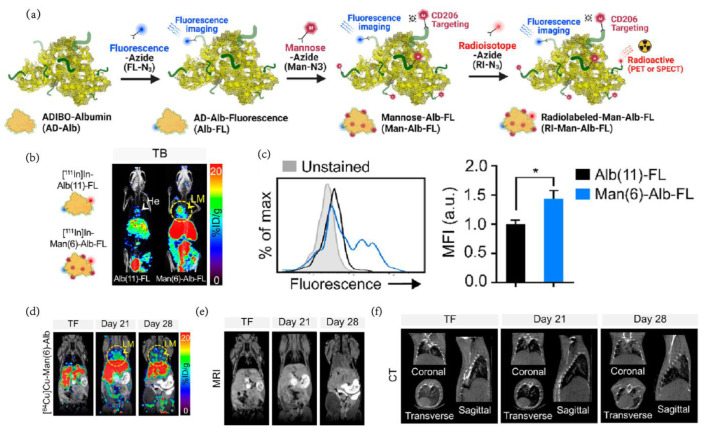
(**a**) Schematic representation of MSAs using click reaction in AD-Alb with other functional molecules. (**b**) SPECT/CT images of 4T1-bearing mice (TB) injected with [^111^In]In-Alb(11)-FL or [^111^In]In-Man(6)-Alb-FL on day 28. (**c**) Fold change in MFI of colabeled FNR-648 on CD^206+^ macrophages from 4T1-bearing mice following injection with Alb(11)-FL or Man(6)-Alb-FL Statistical analysis between groups: (*) *p* < 0.05.(**d**) Representative PET/MRI images of [^64^Cu]Cu-Man(6)-Alb-FL. Representative MRI (**e**) and CT (**f**) images show strong signals from lung metastases on day 28, while no significant change was detected at an earlier stage (day 21) [49]. Copyright 2022, American Chemical Society (Reprinted from ref. [49] under the terms of the Creative Commons CC BY license).

Serving as a powerful tool in clinical and preclinical research because of its high sensitivity and total body penetrance, PET is a molecular imaging technology based on radionuclides [43]. Exhibiting beneficial imaging faculty in atherosclerosis [44], neurodegenerative tauopathies [45], different types of tumors and chronic inflammation [46], PET also showed great potential in targeting TAMs and examining the portrayal of tumor boundaries [47]. TAM-targeting PET nanoprobes are typically constructed by a radionuclide-labeled molecule and a TAM-targeting vehicle. Some receptors mainly expressed by TAMs, such as the colony-stimulating factor 1 receptor (CSF-1R), CD206, scavenger receptors, chemokine (C–C motif) ligand 2 (CCL2), glucan receptor, folate receptors and arginase, usually become the targets of TAM-targeting PET nanoprobes [48]. As a novel imaging nanoprobe with a certain amount of mannose to maximize TAM-targeting ability, Man(6)-Alb is serum albumin containing six mannose molecules created by the click reaction (Figure 6a) [49]. The evidence confirmed that the mannose quantity was derived from the pharmacokinetics of nanoprobes in a healthy mouse model. With six mannose molecule modifications, Man(6)-Alb is regarded as the optimum mannosylated serum albumin (MSA) accumulating in lung metastasis of breast cancer because of its long elimination half-life as unmodified albumin and similar liver uptake as Man(8)-Alb. Capturing images after 19 h (the elimination half-life of Man(6)-Alb) minimizes the impact of signals from the blood pool. More importantly, the similar liver levels between Man(6)-Alb and normal Alb indicate that Man(6)-Alb could instruct metastatic lung lesions of tumor tissue due to the targeting macrophage modification (Figure 6b). The half-life and liver uptake level of Man(6)-Alb enabled it to be competent for the imaging task of lung tumor metastasis. Additionally, in vivo experiments revealed that Man(6)-Alb aggregated around the secondary lesion site. The uptake of the probe by CD^206+^ macrophages was analyzed by flow cytometry. The signal of Man(6)-Alb was approximately 1.4 times that of nonmodified probes (Figure 6c). This article also discusses the application of multimodality imaging, which successfully describes the lung metastasis progression of 4T1 and LLC tumor-bearing mice and obverses tiny metastatic lesions that could not be identified in MRI or CT imaging at 21 days after the operation (Figure 6d–f). Extensive in vivo imaging studies using single photon emission computed tomography (SPECT) and positron emission tomography (PET) have shown that the blood circulation of time-optimized MSA can be used to identify metastatic lesions, and there is a strong correlation between its signal and pulmonary metastasis load.

TAMs had a higher expression level of triggering receptors expressed on myeloid cells (TREMs) than M1 macrophages. Shi et al. [50] established a novel radioligand, ^68^Ga-NOTA-COG1410, for TREM2-targeted PET imaging. By building a macrophage/tumor cell coculture system to obtain tumor-associated macrophages (TAMs), researchers verified the tumor targeting and excellent imaging ability of this nanoprobe. In the orthotopic xenograft CT26.WT tumor model for PET imaging and biodistribution, ^68^Ga-NOTA-COG1410 had excellent affinity and clearly showed colon cancer tumors in situ without inflammatory area signal, which remedies the disadvantage of ^18^F-FDG in discriminating inflammation and tumors. In comparison, the T/NT(target/non-target) of peak tumor uptake (4.22 ± 1.03) of ^68^Ga-NOTA-COG1410 was markedly higher than that of the inflammation area (1.12 ± 0.11). Meanwhile, the high lipophilicity of COG1410 endowed the nanoprobe with BBB penetration characteristics for establishing the latent capacity of intracranial tumor diagnosis. Kim et al. [51] developed a pharmacokinetically optimized, biodegradable ^64^Cu-labeled polyglucose nanoparticle (Macrin) conjugated with L-lysine. Macrin served as a ligand for receptor-mediated cell uptake by phagocytic cells bearing β-glucan receptors (dectin-1 (D1) receptor and complement receptor 3 (CR3)) and exhibited over 90% selectivity to macrophages, indicating great potential to quantify total TAM numbers and their dynamic spatiotemporal distribution in a noninvasive and translationally relevant manner. Compared with low TAM-enriched tumors, high TAM tumors denoted, on average, 730% higher nanomedicine accumulation. For a discussion on radionuclides in PET nanoprobes, please refer to those reviews [52,53].

### 2.4. Near Infrared Fluorescence Dye

**Figure 7:**
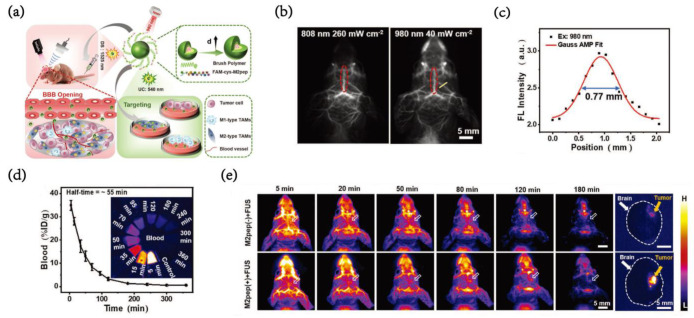
(**a**) Schematic illustration of NIR-IIb EDBM-8.4 nanoprobes for targeted imaging of M2-type TAMs both in vitro and in orthotopic GBM. (**b**) NIR-II images of cerebral vasculature of healthy nude mice after intravenous injection of EDB-8.4 NPs (200 µL, 2.4 mg mL^−1^) under 808 nm (260 mW cm^–2^, LP1500 nm) and 980 nm excitation (40 m©cm^–2^, LP1500 nm), respectively. (**c**) Measuring the vessel line under 980nm excitation. (**d**) The curve of blood circulation half-life plotted from the NIR-II fluorescence intensity of EDB-8.4 NPs in the blood (inset) (mean ± SD, n = 3). (**e**) The NIR-II fluorescence imaging of orthotopic GBM-bearing mice at different times post intravenous injection of EDB-8.4 NPs and EDBM-8.4 NPs (200 µL, 2.4 mg mL^−1^), and the NIR-II fluorescence images of dissected brains after imaging for 120 min [54]. Copyright 2022, Wiley-VCH.

Due to the characteristics of having more potent penetrability than ordinary fluorescence, not involving ionizing radiation or radioactivity and having less interference from the background at a specific wavelength, near-infrared fluorescence imaging has become one of the most useful technologies for TAM-targeting tumor imaging. Zhu et al. [54] structured M2-targeting Er-based NIR-IIb nanoprobes to specifically recognize M2-type TAMs in orthotopic glioblastoma by upconversion fluorescence (540 nm) and downshifting fluorescence (1525 nm) under 980 nm excitation (Figure 7a). A micro vessel with a diameter of 0.77 nm can be clearly observed under 980 nm excitation and has good spatial resolution (Figure 7b,c).

With a blood half-life of 55 min, the targeted probes absorbed by M2 macrophages are approximately 2.5 times those absorbed by M1 macrophages, which provides enough intensity and window for imaging (Figure 7d). After a focused ultrasound (FUS) opened the BBB, M2pep-modified nanoprobes demonstrated extraordinary M2-type TAM-targeting characteristics. Eighty minutes after injection, the tumor background ratio (TBR) reached 4.4, while the nontargeted NP group only reached 3.2. The fluorescence imaging experiment of the brain in vitro showed that the TBR of mice injected with EDBM-8.4 NPs was 11.3, which was much higher than that of the nontargeted EDB-8.4NP group with the 3.0 TBR signal (Figure 7e).

Jiang et al. [55] developed a mannose-adorned NIR/fluorescence dye, Cy7 imaging mark molecule (Cy7-DM). Cy7 SE is one of the most stable dyes with high fluorescence intensity and long wavelength and can be used for in vivo/vitro imaging and real-time monitoring of macrophages in the tumor environment. By covalently binding with dye molecules, simple mannose modification supplied rapid metabolism in nontumor sites and M2 macrophage targeting characteristics, offering a detectable signal in the tumor region 2 h after intravenous injection of Cy7-DM. In the in vivo targeted experiment, the signal intensity of tissues from high to low was as follows: tumor > stomach > lung > spleen > kidney > intestine > brain > liver > inflammatory tissue.

To acquire a better TAM-targeting effect, Nicole et al. [56] manufactured an enzyme-activatable chemokine conjugating nanoprobe (chemo-cat NIR) that was engulfed by TAMs under CCR2 interactions and then evoked NIR through intercellular cysteine cathepsin. The researchers used this enzyme-activatable framework to construct the imaging molecule (chemo-cat NIR) and tumor cell ablation cage prodrug (chemo-cat DOX) and subsequently verified the targeting effect of this preparation on TAMs through the breast cancer E0771-LG mouse model. Chemo-cat NIR can clearly mark TAMs in a lung cancer mouse model. Fluorescent signals from multiple immune cells were analyzed by multiparameter flow cytometry. Chemical-cat NIR clearly labeled TAMs rather than other immune cells, including lung resident macrophages (RMs). Additionally, these biselective probes complement existing antibody drug conjugates and create opportunities to target disease-related immune cell subsets in the tumor microenvironment. Xu et al. [57] introduced a mannose-modified N-azidoacetylgalactosamine (Ac4GalNAz, AAG) liposome, which participated in a two-step click chemistry procedure for detecting TAMs. After entering TAMs, AAG is converted to N-azidoacetylgalactosamine (GalNAz) and tagged mucin-type O-linked glycans. Glycans exist on the surface of TAMs. Then, the azide group of GalNAz combined with DBCO-Cy5 in the next intravenous injection time. The edge of the breast tumor was depicted by fluorescence imaging. As one of the most popular NIR indicator molecules, ICG (Indocyanine green) can also be designed to produce photothermal effects. This nanoprobe explored an imaging model based on a two-step click chemistry procedure that broadens the horizon of noninvasive TAM-targeting imaging. Wan et al. [58] prepared a noncovalent indocyanine green conjugate of C-phycocyanin (CPC@ICG) to target TAM. Compared with free ICG, CPC@ICG improved the photothermal conversion efficiency three times to upgrade tumor suppression and described the tumor spatial information via NIR imaging. Compared with the gradual decline in fluorescence in major metabolic organs, such as the liver and spleen, 8 h after injection, the signal at the tumor site was conserved for more than 24 h and at least twice the signal strength of free ICG at different time periods. Ramesh et al. [59] designed a liposome nanoparticle system synthesized by membrane hydration, encapsulating a diaminofluorescein-2-diacetate (DAF-2-DA) nanoprobe and combining it with a CSF1R inhibiting amphiphile (iCSF1R) to form iCSF1R-NO-NR. iCSF1R-NO-NR responds to nitric oxide levels released from active M1 macrophages, exhibits more than twice the fluorescence signal intensity of the target site than the nontargeted probe and effectively suppresses tumor growth.

Single-walled carbon nanotubes (SCNTs) enable in vivo deep imaging in the NIR-II region [60]. Its optical properties show unique one-dimensional aspects, such as optical transitions between van Hove singularities, large exciton effects and length-dependent plasmon absorptions [61,62,63,64]. Single-walled carbon nanotubes have been used as near-infrared fluorescence imaging molecules for tissue localization [65], surgical guidance [66] and postoperative tumor monitoring [67]. By researching the further mechanism of single-walled carbon nanotubes’ high-authority tumor-targeting ability [68], SCNTs are able to facilitate the progression of circulating monocyte/macrophage phagocytosis, achieving tumor region enrichment [69]. In particular, it has already been proven that a large number of carbon nanotubes can be absorbed by glioblastoma TAMs [70,71]. The uptake of macrophage cells depends largely on the dynamic particle size in CNT dispersions. For large carbon nanotubes, the uptake of macrophages is high [72]. However, relatively small single-walled carbon nanotubes can enhance Toll-like receptor-mediated endocytosis [73,74]. Based on this discovery, CpG oligodeoxynucleotides loaded with SCNT designed by Zhao et al. [75] displayed effective assembly in glioma TAMs. The Cy5.5 signal intensity of the SCNT sCPG group in monocytes and macrophages at the tumor site was more than twice that of the free sCPG group two days after injection. Moreover, SCNT conjugated with ICG mainly enhanced the fluorescence signal of the tumor periphery and was highly valuable in guiding surgeons to assess tumor boundaries [76]. The abovementioned evidence inspired a potential TAM-targeting tumor diagnosis strategy by combining NIR imaging and TAM-targeting casts of SCNT. Thus, SCNT also served as a vehicle candidate for tumor-targeting nanopreparation [77,78].

### 2.5. Ultrasonic Nanobubbles

Nanobubbles have a smaller diameter than microbubbles. Under high sound pressure (>0.1 Pa), the oscillation of bubbles becomes nonlinear, and the volume inflates many times [79]. If the sound pressure exceeds the threshold, the bubbles will violently collapse, which is called inertial cavitation (IC). Based on the abovementioned acoustic behavior, nanobubbles widely serve as ultrasonic contrast agents [80].

Pointing at the overexpression of CSF-1R on the edge of hepatocellular carcinoma TAMs and monocytes, an ultrasonic nanoprobe (NBCSF-1R) conjugated with CSF-1R antibody emerged [81]. With the biotinylation method combining antibody with nanobubbles, NBCSF-1R prolonged the ultrasonic contrast window phase with clear resolution to 30 min in a heterotransplantation hepatocellular carcinoma (HCC) mouse model and insufficient radiofrequency ablation (RFA) mouse model. Studies have assessed the tumor edge rendering ability of NBCSF-1R through immunofluorometric assays, which showed that NBCSF-1R has a high specific targeting capability toward CSF-1R-overexpressing TAMs.

Xiao Sun et al. [82] fabricated a neo-folate-conjugated ultrasonic nanobubble (HA-FOL-NB) loaded with low-molecular-weight hyaluronic acid (LMW-HA) for specific tumor-associated macrophage targeting and repolarization. In ultrasonic imaging, HA-FOL-NB showed outstanding contrast escalation and prominent TAM-targeting ability because of the interaction between folate and folate receptor. The repolarization of macrophages after administration of HA-FOL-NB is the result of downregulation of M2-like macrophage-related factors (Mrc1, CD206, and IL-10) and upregulation of M1-like macrophage-related factors (Nos2 and TNF-α).

**Figure 8:**
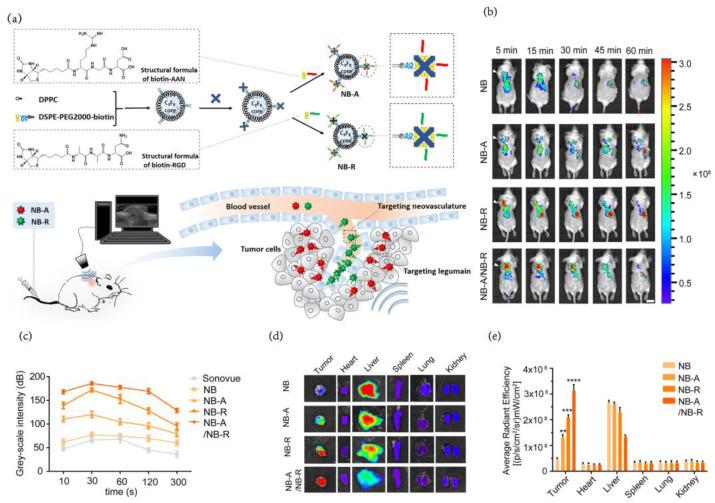
(**a**) Schematic illustration of the fabrication process of AAN-modified NBs (NB-A) and RGD-modified NBs (NB-R) with their application for tumor and neovascular dual-targeted ultrasound imaging of cancer tissue. (**b**) Time course of in vivo fluorescence images of 4T1 mouse breast cancer models injected with DiI-labeled NB, NB-A, NB-R and NB-A/NB-R over time (5, 15, 30, 45, 60 min). (**c**) Corresponding time-intensity curves grayscale ultrasound imaging of tumors after injection of SonoVue MB, NB, NB-A, NB-R and NB-A/NB-R at various time points (10, 30, 60, 120 and 300 s). (**d**) Ex vivo fluorescence imaging of tumors and major organs (heart, liver, spleen, lung and kidney) excised at 30 min postinjection. (**e**) Bar graph showing the integrated intensities of DiI fluorescence in selected organs. A one-way ANOVA test was used for statistical analysis; ** *p* < 0.01, *** *p* < 0.001, **** *p* < 0.0001 compared with the fluorescence intensity of the tumors in the NB group. [83]. Copyright 2022, Elsevier.

With the increasing accuracy of targeting requirements, the double ligand modification strategy was introduced with a powerful targeting effect. By modifying the AAN (tripeptide alanine-alanine-asparagine) peptide (toward legumain, which is highly expressed in breast tumor cells/TAMs) and the RGD (tripeptide arginine-glycine-aspartic acid) peptide (toward tumor angiogenesis) on the surfaces of nanoparticles (Figure 8a), Mi et al. [83] prepared nanobubbles with a small size (~49 nm) by sequential sonication and then purified them using a PD-10 column. NBs were endowed with tumor-targeting ability because of the EPR effect and AAN&RGD peptides, which greatly prolonged the imaging window period. The time intensity AUC of ultrasound imaging in vivo and the fluorescence trend of the tumor site 30 min after injection are both NB < NB-A < NB-R < NB-A/NB-R, while the accumulation content of these probes in the liver is completely the opposite (Figure 8c–e). The fluorescence signal of NB-A/NB-R at the tumor site could still be observed 45 min after injection (Figure 8b).

### 2.6. Multimodal Imaging

Multimodality imaging is widely considered to involve the incorporation of two or more imaging modalities [84]. In multimodality imaging, the need to combine morphofunctional information can be approached by either acquiring images at different times (asynchronous), and fusing them through digital image manipulation techniques, or simultaneously acquiring images (synchronous) and merging them automatically [85]. Choi et al. established murine macrophage Raw264.7 cells expressing enhanced firefly luciferase (Raw/effluc) and murine colon cancer CT26 cells coexpressing Rluc and mCherry (CT26/Rluc-mCherry, CT26/RM) to monitor the migration of TAMs to tumor lesions and the effects of TAMs on tumor progression through bioluminescence imaging (BLI) and fluorescence imaging (FLI) [86]. Bohn et al. performed MRI measurements and PET scans on tumor-bearing mice after intravenous injection with 6.3 ± 0.5 MBq (megabecquerel) ^18^ F-FDG. The superimposed MRI/PET images are used to more comprehensively analyze the uptake of fluorodeoxyglucose by MC38 colon cancer cells and B16 melanoma cells [87]. Foss et al. combined the anatomic imaging capabilities of MRI with the highly sensitive molecular imaging capabilities of PET in an orthotopic syngeneic model of ovarian cancer through [^124^I] iodo-DPA-713, a macrophage-specific imaging agent. By fusing MRI and PET images, specific uptake of the radiotracer was observed within and proximal to the primary tumor at early stages and metastases in the lungs at later stages [88].

The above nanoplatforms for imaging TAMs are presented in Table 1. 

## 3. Principlesof Designing TAMs Imaging Nanoprobes

### 3.1. Targeting Efficiency

Currently, targeted ligands range from peptides, antibodies and oligonucleotides to cell membranes, exosomes, etc. In this section, we discuss the targeting effect of different TAM-targeting agents from a materials perspective.

The following table summarizes the targeting efficiency of nanoprobes presented in this review. Mannose-, dextran- and M2-targeting polypeptides are the mainstream TMAs targeting refinement, which remarkably improves imaging performance. Notably, the double ligand strategy combined with AAN and RGD peptides shows more extraordinary targeting ability than single modified probes. This indicates that the next step of upgrading TAM-targeting efficiency may be exploring targets to synergistically enhance targeting capability and designing double selection methods that vary by TAM type.

In the continuous development of TAM-targeting approaches, the strategy that macrophage exosomes serve as carriers has gradually become of interest. From the perspective of the tumor microenvironment niche, exosome-encapsulated noncoding RNAs (ncRNAs) are the interaction medium between tumor cells and TAMs [96]. Some tumor-derived exosomes can paradoxically promote M1 macrophage polarization. Exosomes containing miR-130, miR-33 [97] or miR-125b-5p [98] can induce macrophages to transform into the M1 type and activate antitumor effects.

Gunassekaran et al. [99] reported IL4R peptide-modified M1 macrophage exosomes (IL4R-Exo(si/mi)), which engulfed NF-κB p50 siRNA and miR-511-3p to target M2 macrophages and reduce the expression of related genes. With high tumor-targeting distribution in the 4T1 mouse model, IL4R-Exo(si/mi) not only exhibited a better tumor-targeting effect than Exo(si/mi) but also showed tumor accumulation even higher than liver accumulation 2 h after injection (tumor:liver ≈ 3.3:1.3). NF-κB p50 siRNA knocks down NF-κB p50, while miR-511–3p downregulates genes involved in the protumoral activity of TAMs and inhibits tumor growth. Kamerkar et al. [100] described engineered exosomes with antisense oligonucleotides (ASOs) that selectively silence STAT6 in TAMs. Exosomes were more effective in delivering ASOs to bone marrow cells in CT26 subcutaneous tumors (TAMs, DCs, and MDSCs were 1.3-, 3.5-, and 5-fold higher than free ASOs, respectively).

Simultaneously, the strategy of biomimetic membrane coating could effectively target TAMs. In the preparation of a selectively targeting and polarizing TAM biomimetic polymer magnetic nanocarrier (PLGA-ION-R837@M (PIR@M)) designed by Liu et al., PLGA-ION-R837 (PIR) NPs loaded with oleic acid-modified Fe_3_O_4_ NPs (ION), and R837 were first fabricated via emulsification and solvent evaporation methods [101]. Then, after several extrusions through a 200 nm polycarbonate membrane, M1 macrophage membranes from LPS-treated macrophages served as the coating of PLGA-ION-R837 (PIR). With Fe_3_O_4_ NPs activating the IRF5 pathway and R837 evoking the NF-κB pathway, PLGA-ION-R837@M (PIR;@M) synergistically enhanced macrophage repolarization. Regarding the uptake of M2 macrophages, the fluorescence intensity of the PC@M group was 1.87-fold higher than that of the PC group after 2 h of incubation. The signal of NPs in M2 macrophages was significantly higher than that in 4T1 tumor cells. A hemoglobin–poly(ε-caprolactone) (Hb–PCL) conjugate self-assembled biomimetic nano red blood cell (nano-RBC) system was designed to target and eliminate M2 TAMs by the CD163 receptor and DOX, which had a great M2 macrophages targeting effect (tumor site absorbs fluorescence signal M2:M1 ≈ 7:2) [102]. The information of targeting efficiency is presented in Table 2.

### 3.2. Pharmacokinetics

Pharmacokinetics are vital to TAM-targeting imaging nanoprobes. It not only impacts the imaging window time and resolution but also considerably affects the targeting ability and biosafety of the nanoprobe. When designing a nano imaging probe, people must consider the balance of organism clearance and targeting capacity. Imaging agents are always concentrated in the liver and kidney after entering the internal environment, which usually reduces the targeting efficiency of instructors and causes a high imaging background and unclear signal at the tumor site. Meanwhile, it is more difficult to image tumors in metabolic organs. Promoting rapid probe clearance in the nontumor region may be a promising strategy for improving imaging contrast. To probe the liver and kidney enrichment problem, Luo et al. [90] developed dextran-indocyanine green (DN-ICG) nanoprobes for the dynamic imaging of TAMs in pancreatic cancer. DN-ICG exhibited a 279% NIR-II fluorescence intensity compared to that of free ICG, gradually metabolized in the liver and remained in the pancreatic tumor, achieving a high signal-to-background ratio (SBR = 7) in deep tissue.

Different imaging technologies have different requirements for TAM-targeting approaches. MRI and PET/CT nanoprobes usually have relatively stable imaging capabilities at the atomic and inorganic compound levels. More attention should be given to the biological safety exploration of MRI/PET nanoprobes, especially in the aspects of internal deposition and radioactive safety [104]. Currently, researchers have partially achieved the abovementioned goals through preparation forms or targeting methods such as PEG modification and macrophage-targeted ligand modification.

However, NIR/fluorescence probes conjugating structures are delicate, resulting in a lower imaging effect after swallowing by TAMs. Therefore, this type of probe is more suitable for the strategy of achieving similar targeting to TAMs while avoiding phagocytosis of TAMs. Recently, studies on macrophage membranes and exosome preparation have increased. In light of the interaction between TAMs and cancer cells, macrophage membrane-coated upconversion nanoparticles (MM-UCNPs) were fabricated and generated an intense signal 48 h after MM-UCNPs injection when there was no background signal for the rest of the mouse body except for the tumor region [105]. Macrophage membrane coating endowed the nanoprobe with significantly enhanced blood retention and tumor-targeting ability. Engineered exosomes can also target tumor sites and efficaciously improve the effect of NIR imaging [106]. Fan et al. [107] tried to avoid limited exosome production by squeezing M1 macrophage exosomes (M1mv) with a pneumatic liposome extruder. With the Cy5.5 and BHQ3 double NIR probes marked, M1mv and DOX were used to simultaneously explore their antitumor effects. Twenty-four hours after injection, in vivo imaging showed a clear optical signal with a negligible background signal, implying that macrophage exosomes have great latent capability in tumor imaging.

Continuing to deeply focus on the idea of improving the biocompatibility of imaging agents, we find that taking macrophages themselves as carriers of imaging probes can endow the probe with the ability to target tumors and avoid phagocytosis. For example, Wang et al. [103] fabricated a macrophage-platform photothermal nanoprobe loaded with MFe_3_O_4_-Cy5.5, which allowed fluorescence, photoacoustic and magnetic resonance imaging of tumor tissue. Initially, this nanoprobe differentiates tumors from normal tissue according to the characteristics of macrophage aggregation at the edge of glioma. Then, surgical resection of the tumor was guided. At the same time, this nanoprobe can induce effective photothermal therapy to inhibit tumor recurrence. This macrophage-mediated imaging probe exhibited better biocompatibility and retention with a 5.62 h blood half-life and 8, 11, 15 and 14% tumor site percentage injected dose per gram of tissue (%ID/g) at 1, 3, 5, and 7 days, respectively. Five days after injection, MFe_3_O_4_-Cy5.5 gradually surrounded the tumor region without a measurable signal decline, while nonmembrane coating particles refused to target the tumor and were eliminated piecemeal.

A macrophage-mediated strategy also applies to MRI nanoprobes. Loading hydrophilic Fe@Fe_3_O_4_ nanoparticles with a core-shell structure into RAW264.7 cells, researchers acquired an excellent T2-weighted MRI contrast agent with photothermal performance [108,109]. Regarding gold nanoparticles, macrophage-mediated delivery of small gold nanoparticles for tumor hypoxia photoacoustic imaging [110] and dendrimer-entrapped gold nanoparticles for orthotopic osteosarcoma CT imaging were also explored [111]. Moreover, macrophage (MA)-laden gold nanoflowers (NFs) embedded with ultrasmall iron oxide nanoparticles (USIO NPs) were created to enhance CT and MR imaging of tumors [112].

### 3.3. Surgical Operation Guidance

Although research on TAM-targeting nanoprobes is blooming with diversified imaging methods, targeting approaches and preparation forms, imaging agents that can be applied to clinical tumor patients are still limited. Hitherto, surgery is still the most effective treatment for early solid tumors. Thus, in view of surgery guidance, doctors at the frontline of the clinic urgently require a convenient, economical and effective tumor-indicating probe to eliminate the cumbersome protective suit for CT-guided surgery.

**Figure 9:**
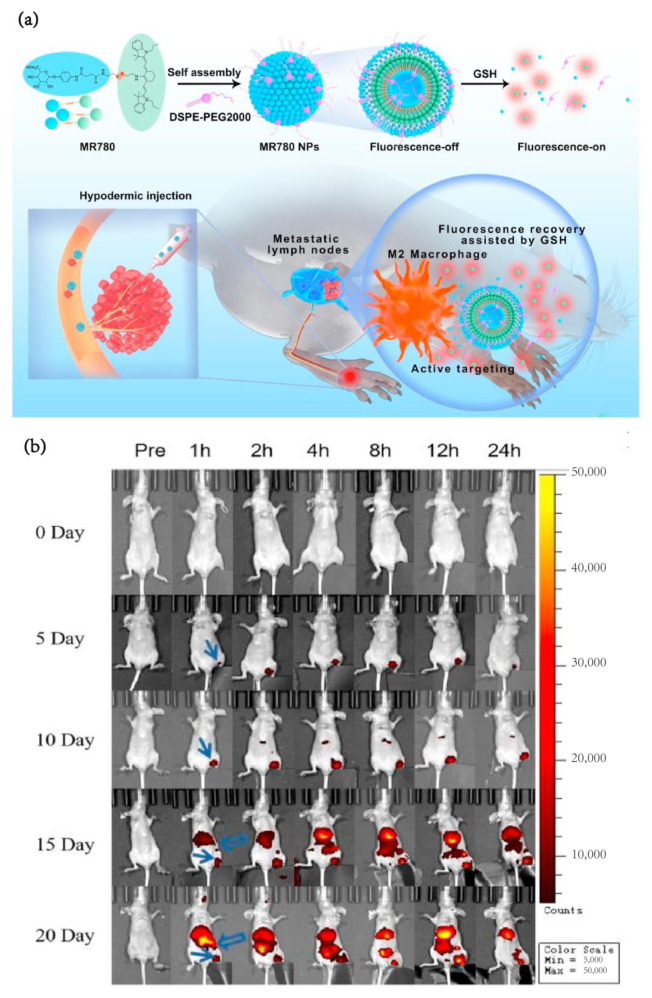
(**a**) Schematic diagram for the fabrication and function of MR780 NPs: 4-aminophenyl α-d-pyranoside and IR780 was connected via cystamine to obtain MR780 conjugate, and then self-assembled with DSPE-PEG2000 to prepare MR780 NPs. (**b**) Fluorescence imaging of lymph nodes in normal mice and plantar 4T1 tumor-bearing mice every 5 days [91]. Copyright 2022, Elsevier.

In surgery guidance, compared with visible fluorescence, near-infrared fluorescence imaging technology is more widely used in cancer surgery guidance because of its penetration depth, low imaging background and the simplicity of detection methods [113]. Surgery guided by NIR can result in better differentiation of the lesion site, more optimized anesthesia time and a lower false positive rate of the normal tissue at the incision edge [114]. NIR imaging exhibits a much more influential potential than MRI and normal visible fluorescence. In the diagnosis of lymph node micrometastasis in breast cancer, a frequently used method (i.e., ultrasonic imaging) was confused by poor resolution and contrast agent spillover. To solve this problem, researchers [91] conjugated mannose with IR780, a near-infrared fluorescence molecule, through cystamine. Then, the coupling compound self-assembled into an M2 macrophage-targeted fluorescent probe (MR780 NPs). Mannitol binds to the CD206 receptor on the surface of TAMs and promotes the internalization of TAMs into nanoparticles. Upon reaching the tumor microenvironment, MR780 disassembled and recovered the IR780 signal. MR780 NPs showed a highly sensitive response to early breast cancer with lymph node metastasis and distant metastasis (Figure 9a). When metastatic lymph nodes were only 2 mm, the fluorescence signals of obvious metastasis could be captured by MR780 NPs (Figure 9b). Furthermore, MR780 NPs instructed the removal of metastatic lymph nodes without harming normal lymph nodes, which produced a new method to evaluate lymph node micrometastasis in breast cancer.

**Figure 10:**
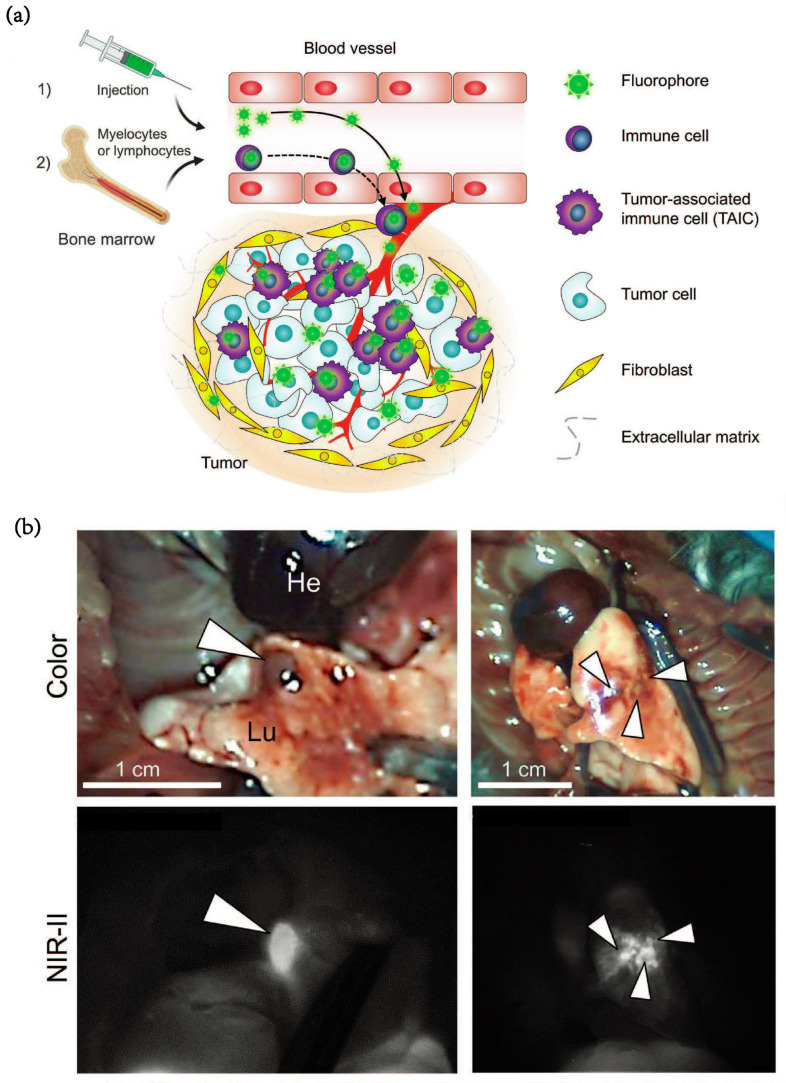
(**a**) Schematic illustration for two-step mechanism of tumor-targeted SH1 fluorophore. (**b**) 50 nmol of SH1 was injected into the mouse model of orthotopic lung cancer tumors 48 h prior to imaging. The white arrowheads indicate lung tumors. (excitation = 808 nm; power density = 30 mW cm^−2^; exposure time = 25 ms; optical filter = 1070 nm LP) [92]. Copyright 2022, Wiley-VCH.

Kang et al. [92] designed TAIC targeting fluorescence (SH1) as an NIR-II imaging probe that can track almost all types of tumor region immune cells. Synthesized by a condensation reaction between the heptamethine core and heterocyclic indolium salt, SH1 endowed TAIC with targeting ability without additional ligand modification. After intravenous injection, fluorescence signals from the small tumors were clearly shown in a real-time image (Figure 10a). They also confirmed that the edge of the anatomical tumor can be distinguished by the strong fluorescence signal of SH1 (Figure 10b) and has a good correlation with the results of H&E staining.

In regard to tumors with aggressive invasiveness and diffusion ability, surgical resection of the tumor region requires an extremely accurate tumor margin definition [93]. One of the significant characteristics of glioblastoma multiforme (GBM) is abundant macrophage infiltration. To further guide the intraoperative staging and radical surgery of GBM, Lee et al. [93] introduced an NIR-fluorescent nanoparticle (NF-SION) that used hydrophobic iron oxide nanoparticles as the core and a silica layer as the coating. With high water dispersion capacity and robust fluorescence stability, NF-SIONs are enriched in TAMs because of their high endocytosis activity and exhibit a peak NIR signal 24 h after injection, which profiles the tumor boundary for surgery.

Despite having so many powerful advantages, TAM targeting NIR imaging technology is still confronted with inconvenience from additional imaging viewing equipment. Because external instruments are often used to intervene in the tumor site during surgery, the penetration depth limit of visible light becomes less lethal, and the advantage that no other equipment required for visual observation is further magnified during operation. Fluorescent silica nanoparticles (SiNPs) with a diameter range of 200–1000 nm selectively accumulate in the TAMs of abdominal metastatic ovarian cancer in a murine model [115]. Marotta et al. [94] combined upconverting nanoparticles (UCNPs) with SiNPs, generating a visible light signal by NIR excitation to enhance the tumor boundary profile in real-time surgical resection and provided a simple and feasible imaging scheme for operation guidance. Image analysis showed that compared with vision-based surgical resection, the number of residual tumors after image-guided surgery was significantly reduced. Approximately 10.7% of the original tumor signal was retained after naked eye surgery, while only 0.2% of the original tumor signal was retained after UCNP-guided detection.

**Figure 11:**
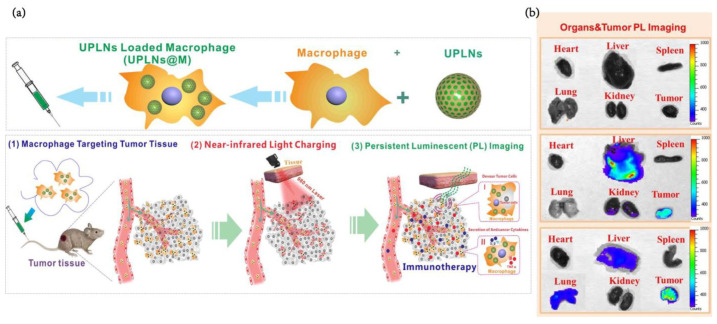
(**a**) Schematic diagram indicating applications of near-infrared (NIR) excitation upconverting persistent luminescent nanoparticles (UPLNs)-loading macrophages for real-time tracking of tumor therapeutic macrophages in vivo after endocytosing these nanophosphors in vitro and following macrophage biodistribution by simple whole animal optical detection. (**b**) After tail vein injection for 24 h, the distribution of persistent luminescence nanophosphors in excised organs and tumors for different treatment groups [96]. Reprinted (adapted) with permission from [96]. Copyright 2018, American Chemical Society.

Additionally, Yb^3+^ could absorb more than one NIR low-energy pump photon, while Tm^3+^ would turn them into high-energy photons with shorter wavelengths [116]. Using mesoporous silica as a vehicle and coating the macrophage membrane, upconversion persistent luminescent nanoparticles excited by 980 nm near infrared (UPLN) holding Y^3+^, Yb^3+^ and Tm^3+^ exhibit robust visible fluorescence signals 40 min after excitation (Figure 11a) [95]. Compared with the UPLNs group, the UPLN-loaded macrophage group had a high persistent luminescence intensity in the tumor area (Figure 11b). Due to the feature of tracking after being engulfed by TAMs, UPLN may be suitable for some situations of ultralong-term in vivo monitoring and surgical environments.

## 4. Summary and Future Directions

TAMs, which generally accumulate at the edge of tumors, play an important role in cancer progression and the tumor immune microenvironment. As a consequential scope to unveil tumor status and limit the boundaries of tumors, TAM-targeting nanoprobes have received increasing attention and focus. This review mainly introduces some materials applied to target TAMs and focuses on the literature on TAM-targeting efficiency, pharmacokinetics and surgical guidance. In addition to exploring the application of archetypal imaging molecules such as iron oxide, ^19^F and nanobubbles, some novel materials, such as single wall carbon nanotubes, gold nanoparticles and manganese imaging agents also take part in this examination of TAM-targeting probes.

Some tendencies have appeared in the process of designing TAM-targeting nanoprobes at the current stage, including: 1. Combining diagnosis with therapeutic treatment. 2. Noteworthy interest in multimode and multilevel imaging probes. 3. Requiring high-precision target ability. 4. Refining the pharmacokinetics of probes to achieve a low background signal and distinct tumor site imaging. Additionally, pharmacokinetic improvement will prolong the imaging window of TAM-targeting probes in a serviceable way and provide long-term noninvasive tumor monitoring platforms. Simultaneously, TAM-targeting imaging technology profoundly interacts with other technologies as well. According to the PyRadiomics workflow, Starosolski et al. [117] analyzed 900 radiomic features in the quantitative image feature pipeline (QIFP) to distinguish high-TAM tumors from low-TAM tumors via machine learning, which is a difficult task for normal CT imaging.

However, there are still some obstacles hindering TAM-targeting imaging strategies. Initially, TAM-targeting tumor imaging was not a direct way to characterize cancer cell appearance. Regardless of the great imaging effect coming from phagocytosis and the critical niche of TAMs in the TIME, there still exists an information feedback chain from macrophages to tumor cells. In the mode of estimating the preparation therapy effect and guiding operation, the information feedback chain may be swayed by fluctuations in the immune environment and inflammation-related factors (e.g., pseudo progression of tumors created by the transformation of the local inflammatory environment during the radiotherapy/chemotherapy cycle [118] and changes in TAM targeting the imaging effect caused by temperature alteration of magnetotherapy/photothermal therapy [119]). These problems arising from specific applications constantly put forward new requirements for the design of targeted TAM probes. Furthermore, the practical function and components of TAMs are altered with tumor type and tumor process. Currently, TAM-targeting probes are mostly a combination of a single ligand and imaging molecules, which can neither accurately provide the specific phenotype distribution of TAMs nor precisely apply to different types of tumors. Therefore, it is still a heavy task to discern the exact phenotypes of macrophages. Eventually, targeted TAM imaging strategies may be limited by the number and poor invasiveness of TAMs in some tumor application scenarios. However, the imaging ability of TAM-targeted probes can be improved by further combining the regulation of the immune microenvironment and recruitment of macrophages at the tumor site. Furthermore, there are still some difficulties on the path from preclinical to clinical application of TAMs-targeting probes. Because the conditions of animal models and some murine tumor immune microenvironments are different from those of the clinic, the pharmacokinetic performance of targeted TAMs probes may be unexpressed. Increasing the exploration of large animal models in subsequent studies to get close to the real situation of humans and paying more attention to immune background analysis may be acceptable choices. Meanwhile, MRI and PET imaging technology is more widely used in clinical practice. The preparation of corresponding probes is relatively simple, safe, effective and repeatable. The clinical transformation difficulty of imaging probes targeting TAMs in PET and MRI is less than that of NIR imaging and MPI.

## Figures and Tables

**Table 1:** Nanoplatforms for imaging TAMs.

Number	Material	Type of NPs	Disease	Cell line	Immune Background	Animal Model	Administration	Dose	Imaging Methods	Targeting Approaches	Model	Reference
1	Iron oxide	Iron oxide nanoparticle	Glioblastoma	MbTIC0309 brain tumor cells	immunocompetent	8–10 weeks Female C57BL/6 mice	i.v.	30 mg/kg	MRI	Phagocytosis	in vivo	[17]
2	Iron oxide	Mannose-PEG(Poly(ethylene glycol))-b-AGE polymer coated iron oxide nanoparticles	Breast cancer	4T1 tumor cells	immunocompetent	6–8 weeks old female Balb/C mice	i.v.	5 mg Fe/kg	MRI	Mannose modified	in vivo	[18]
3	Iron oxide	M2 macrophage targeted peptide functionalized superparamagnetic iron oxide	Breast cancer	4T1 tumor cells	immunocompetent	female BALB/c mice	i.t. (in situ)	0.8 uL mm^−3^ of tumor volume, 10 UG uL^−1^ *	MRI	M2 macrophage targeted peptide	in vivo	[19]
4	Iron oxide	Comprising superparamagnetic iron oxide nanocrystals and nitric oxide (NO) donors	Pancreatic tumor	Panc02 tumore cells	immunocompetent	4–5 weeks old, female C57BL/ 6J mice	i.v.	2 MG/mL,100μL	MRI	Phagocytosis	in vivo	[22]
5	Iron oxide	Sulfated-dextran coated iron oxide nanoparticles	Breast cancer	4T1 tumor cells	immunocompetent	6–12 weeks old female C57BL/6 mice	i.v.	15 mg Fe/kg 30 mg Fe/kg	MRI	Dextran	in vivo	[23]
6	Mn^2+^	Bioactivated in vivo assembly magnetic resonance probe	Breast cancer	MDA-MB-231 tumor cells	immunosuppression	Female BALB/C nude mouse	i.v.	20 mg/kg	MRI	Mannose modified	in vivo	[26]
7	MnO_2_	HA-coated, mannan-conjugated MnO_2_ particle	Breast cancer	4T1 tumor cells	immunocompetent	Female Balb/c mice	i.v.	Man-HA-MnO_2_ NP (13.2 mg/kg) Dox (5 mg/kg)	MRI	Mannose modified and Hyaluronic acid coated	in vivo	[28]
8	MnO_2_	Hyaluronic acid (HA) and PLR-coated manganese dioxide (MnO_2_) nanoparticles	Sarcoma	S-180 murine sarcoma cancer cell line	/	/	/	/	MRI	Hyaluronic acid and poly(L-arginine) modified	in vitro	[29]
9	MnO_2_	An upconversion nanoparticle (UCNP) as the core, hollow mesoporous silica wrapped on the outside of the UCNP with DOX filled within the cavity, MnO_2_ nanosheets modified in mesopores as hypoxia-sensitive agents	Cervical carcinoma	Hella cells	immunosuppression	BALB/C nude mice	i.v. and i.t.	8 MG/mL, 100 μL	MRI(T1)	Phagocytosis	in vivo	[30]
10	^111^In	Optimization of mannosylated serum albumin (MSA)	Breast cancer/Melanoma	4T1/B16F10/LLC celmmuneuno-competent	7–8 weeks old female BALB/c mice and C57BL/6 mice	i.v.	11.1 MBq (megabecquerel)	PET	Mannose modified		in vivo	[49]
11	^68^Ga	^68^Ga-NOTA-COG1410	Colon cancer	PCM/MGC-803/AGS/CT26.WT celmmuneuno-competent and immunosuppression	8 weeks old male BALB/c nude mice (18–20 g) and male BALB/c mice	i.v.	1 μg, 100 μL	PET	COG1410 for TREM2 targeting		in vivo	[50]
12	^68^Ga	[^68^Ga]Ga-DOTA-M2pep	Melanoma	B10-F10 cells	immunocompetent	6–8 weeks old male C57BL/6 mice	i.v.	3.7 MBq per mice	PET	M2pep modified	in vivo	[89]
13	^64^Cu	^64^Cu-labeled polyglucose nanoparticle	Breast cancer/Lung tumor/Colon	4T1/KP1.9/MC38 cells	immunocompetent	7–12 weeks old C57BL/6 mice and 6–8 week old BALB/c mice	i.v.	8.5 ± 2.4 MBq in 150 ± 10 μL PBS	PET	Polyglucose modified	in vivo	[51]
14	Er-based probe	M2-targeting Er-based NIR-IIb nanoprobes	Glioblastoma	U87MG glioma cells	immunosuppression	6–8 weeks old specific pathogen-free (SPF) grade female nude mice	i.v.	2.4 MG/mL, 200 μL	NIR	M2pep modified	in vivo	[54]
15	Cyanine 7	Deoxymannose labeled cyanine 7	Hepatoma	SMMC-7721 human hepatoma cells	immunosuppression	Nude mice	i.v.	2 nmol, 100 μL	NIR	Deoxymannose	in vivo	[55]
16	Sulfo-Cyanine 5	Enzyme-activatable chemokine conjugates nanoprobe (chemo-cat NIR)	Breast cancer	E0771-LG celmmuneuno-competent	C57BL/6 mice	i.v.	0.3 nmol per mouse	NIR	Enzyme-activatable chemokine conjugates		in vivo	[56]
17	Indocyanine green(ICG)	Noncovalent indocyanine green conjugate of C-phycocyanin (CPC@ICG(Indocyanine green))	Cervical carcinoma	H22 cells	immuno-competent	Kunming (KM) mice	i.v.	1.25 mg ICG/kg	NIR	C-phycocyanin modified	in vivo	[58]
18	Diaminofluorescein-2-diacetate (DAF-2-DA)	NIR-NO nanoprobe combined with CSF1R inhibiting amphiphile	Breast cancer	4T1 cells	immuno-competent	BALB/c mice	i.v.	2 mg NO-NR/kg	NIR	CSF1R inhibiting amphiphile	in vivo	[59]
19	Nanobubble	CSF-1R targeted nanobubble	Hepatocarcinoma	SMMC-7721 cells	immunosup-pression	5–6-week-old male BALB/c nude mice	i.v.	4 × 10^7^ NB CSF-1R per mice	Ultrasound imaging	CSF-1R-conjugated	in vivo	[81]
20	Nanobubble	Folate-conjugated ultrasonic nanobubble (HA-FOL-NB)	Lung carcinoma	Lewis lung cells	/	/	/	/	Ultrasound imaging	Hyaluronic acid modified	in vitro	[82]
21	Nanobubble	AAN peptide and RGD peptide modified nanobubbles	Breast cancer	4T1	immuno-competent	4–5 weeks old BALB/c mice	i.v.	200 μL NPs per mice	Ultrasound imaging	AAN peptide and RGD peptide	in vivo	[83]
22	Gold nanoparticle	PGMP-small interfering RNA (siRNA) nanoparticles	Non-small cell lung cancer	A549 cells	immunosup-pression	BALB/c nude mice	i.v.	3 mg/mL, 100 μL	Ultrasound imaging	Phagocytosis	in vivo	[32]
23	Gold nanoparticle	Glycol-chitosan-coated gold nanoparticles (GC-AuNPs)	Delivering Tumor antigen to lymph nodes	/	immuno-competent	5 weeks old female healthy nu/nu mice	injected through the right side of the tongue	2.5 mg Au/mL, 80 μL	PA	Phagocytosis	in vivo	[33]
24	Cyanine 5/Iron oxide	Mannose-targeted liposomes (MAN-lipo-AAG) liposomes (lipo-AAG) encapsulating Ac4GalNAz	Breast cancer	4T1 cells	immuno-competent	6 weeks old female BALB/c mice	i.v.	50 mg/kg	NIR	Mannose modified	in vivo	[57]
25	Silver nanoclusters	AgNCs in combination with M1-like macrophages (namely the MAC@NC complex)	Lung metastasis breast cancer	4T1-LG12 cells	immuno-competent	7–8 weeks old female BALB/c mice	i.v.	2.0 × 10^6^ MAC@NC per mice	NIR	Macrophage vehicle	in vivo	[35]
26	Fluorine-19	Fluorine-19(^19^F)@PLGA-PEG-Man@perfluoro-15-crown-5-ether (PFCE)	Breast cancer	4T1 cells	immuno-competent	6–8 weeks old BALB/c mice	i.v.	7.01 × 10^19^ ^19^F per mice	MRI	Mannose modified	in vivo	[39]
27	Fluorine-19	Perfluorocarbon-containing nanoparticles (PFC-NP)	Gliomagenesis, breast-to-brain metastasis, and breast cancer	RCAS-PDGFB-HA and PDGFB-HA-SV40-GFP DF1 cells	immuno-competent	6–7 weeks old C57BL/6J mice	i.v.	10 Gy per mice	MRI	Phagocytosis	in vivo	[42]
28	Indocyanine green	Dextran-indocyanine green	Pancreatic cancer	SW1990 pancreatic cancer cells	immunosup-pression	Female BALB/c nude mice	i.v.	0.5 ICG mg/kg	NIR	Dextran	in vivo	[90]
29	IR780	Self-assembled IR780 conjugated with mannose	Lymph node metastasis breast cancer	4T1 cells	immuno-competent	5 weeks old female BALB/c mice	subcutaneous injection (s.c.)	2 mg/kg	NIR	Mannose modified	in vivo	[91]
30	Heptamethine cyanine-based fluorophores	TAIC targeting heptamethine cyanine-based fluorophores	Pancreatic, breast, and lung cancer	Pan02,E0771 and LLC cells	immunosup-pression and immunocompetent	8 weeks old C57 BL/6 mice	i.v.	1 μmol/kg	NIR	Phagocytosis	in vivo	[92]
31	Cyanine-5.5	Near-infrared fluorescent silica coated iron oxide nanoparticles	Glioblastoma multiforme	U87-MG cells	immuno-competent	6 weeks old male BALB/c nude mice	i.v.	200 μg Fe	NIR	Fluorescent silica coated	in vivo	[93]
32	UCNP	Combined up-converting nanoparticles(UCNPs) with silica nanoparticles	Ovarian cancer	OVCAR8-GFP cells	immunosup-pression	7 weeks old athymic nude mice	i.p.	1.37 × 1010 UCNPs in 1 mL PBS	Visible fluorescence	Phagocytosis	in vivo	[94]
33	UCNP	Based on (Zn_2_SiO_4_:Mn):Y^3+^, Yb^3+^, Tm^3+^ upconverting persistent luminescent nanophosphors	Melanoma	B16 cells	immuno-competent	8 weeks old C57/B6 mice	i.v.	0.5 mg/mL, 100 μL	Visible fluorescence	Phagocytosis	in vivo	[95]

* 0.8 uL mm^−3^ of tumor volume, 10 ug uL^−1^ means 0.8 μL (10 μg/μL) of probes was given to every cubic millimeter of tumor.

**Table 2:** Targeting efficiency of TAM-imaging nanoprobe.

Imaging Material	Tumor Type	Imaging Methods	TAMs Targeting Approaches	Targeting Effect	Reference
Iron oxide	Breast cancer	MRI	Mannose modified	Colocalization degree of target protein 6 h: Targeting: non-targeting = 93.5%: 57.3%	[18]
Iron oxide	Breast cancer, 4t1 model	MRI	M2 targeting peptide	In vitro targeting 6 h: Targeting: non-targeting = 185%	[19]
Iron oxide	Pancreatic cancer	MRI	/	Tumor signal in 48 h: Targeting (5 × 10^3^ a.u.) Non-targeting (2 × 10^3^ a.u.)	[22]
Iron oxide	Breast cancer, 4t1 model	MRI	Mannose modified	Tumor signal in 24 h: Targeting (3 × 10^8^ p/s/cm^2^/sr) Non-targeting (1.3 × 10^8^ p/s/cm^2^/sr)	[28]
Near-infrared dye cyanine 7 (Cy7)	Hepatoma cell	NIR	Deoxymannose	Tumor signal in 8 h: Targeting (42 × 10^8^ p/s/cm^2^/sr)	[55]
^111^In	Breast cancer, 4t1 model	PET	Mannose	Lung metastasis %ID/g in 24 h: Targeting (5) Non-targeting (2)	[49]
^68^Ga	Gastric cancer	PET	COG1410, as a ligand of TREM2	Tumor %ID/g in 2 h Targeting (6)	[50]
^64^Cu	Breast cancer 4T1 mice model	PET	Polyglucose	Tumor %ID/g in 24 h Targeting (10)	[51]
Er-based rare-earth nanoparticles	Orthotopic Glioblastoma	NIR	M2pep polypeptide	In vitro targeting: Targeting: non-targeting = 207%	[54]
Indocyanine green	Hela human cervical cells	NIR	C-phycocyanin	In vitro targeting: Targeting: non-targeting = 330%	[58]
Nano bubble	Breast cancer 4t1 mice model	ultrasound imaging	AAN peptide and RGD peptide	Tumor signal in 0.5 h: Targeting (3.2 × 10^8^ p/s/cm^2^/sr) Non-targeting(0.5 × 10^8^ p/s/cm^2^/sr)	[83]
Indocyanine green	Pancreatic Cancer	NIR	Dextran	Tumor signal in 24 h NIR-I (90 a.u.) NIR-II (130 a.u.)	[90]
Heptamethine-cyanine-based fluorophores	Lung carcinoma, pancreatic ductal adenocarcinoma (PDAC), and triple negative breast adenocarcinoma	NIR	/	Fluorescence positive rate of cells co-cultured with preparation: SH1:free ICG = (45.7%: 1.2%)	[92]
Cyanine-5.5	Glioblastoma	NIR	Fluorescent silica coated	Tumor signal in 24 h: Targeting (21,000 a.u.)	[93]
Cyanine-5.5/Iron oxide	Glioblastoma	NIR/MRI	Macrophages membrane coating	Tumor %ID/g in 24 h: Targeting (8) Tumor %ID/g in 7 days: Targeting (14)	[103]

## Data Availability

Not applicable.
